# Supplementary material for: The Effects of Green Exercise on Physical and Mental Wellbeing: A Systematic Review
Source: Int J Environ Res Public Health. 2019 Apr 15;16(8):1352. doi: 10.3390/ijerph16081352 (PMC6518264; doi:10.3390/ijerph16081352)
Supplement: Supplementary file 1 [file ijerph-16-01352-s001.pdf]

## Supplementary Files

**Table S1.** Characteristics of studies

| Study (country) | Design and Sample | Green Exercise Conditions | Non-Green Exercise Conditions | Outcomes (measure) |
|---|---|---|---|---|
| Byrka & Ryczko 2018 (Poland) | **Design:** RCT<br>Acute<br>**Sample:** 64 dancers (28, outdoor group; 36, indoor group) with least 3 months of salsa experience (80% female, 20% male).<br>Age (mean ± SD): 29 ± 9 y | 40-min of outdoor salsa dancing at moderate-vigorous intensity<br>**Description:** "park condition" in Southern Park in Wroclaw. "The area was surrounded by a knee-high wall and old trees on two sides."<br>**Temperature:** NR | 40-min of indoor salsa dancing at moderate-vigorous intensity<br>**Description:** "dance room condition… 30 square meters in size, with 4-meter-high ceilings, mirrors on one wall and windows on the other"<br>**Temperature:** NR | Affect (combined positive emotions via EFI); Physical activity (via accelerometers); Physical exhaustion (via EFI) |
| Calogiuri 2018 (Norway) | **Design:** non-RXT (outside trial first, random order of other conditions)<br>Acute, 3 sessions separated by at least 15-min<br>**Sample:** 26 healthy university students and employees (46% female, 54% male).<br>Age (mean ± SD): 26 ± 8 y<br>BMI (mean ± SD): 23.1 ± 5.0 kg/m$^2$ | 1) 10-min self-paced walk outdoors in a natural environment.<br>**Description:** "a paved trail by a river, with view of grassland and some trees"<br>**Month:** May<br>**Temperature:** 7-17°C<br>2) 10-min self-paced walk on a manually driven treadmill, with VR goggles and headphones reproducing a 360-video and sound of the same walk as above (1).<br>**Temperature:** 21°C. | No non-green exercise intervention included. | Affect (PAAS); Effort (6-20 Borg RPE scale); Enjoyment (1-item scale); HR; Perceived potential for restoration (PRS); Walking speed (outdoors: GPS, Indoors: treadmill computer); Presence and cyber sickness (VR only). |
| Calogiuri 2015a (also 2015b) (Norway) | **Design:** RCT<br>Brief longitudinal, 2 sessions with 2 week and 10 week follow-up<br>**Sample:** 14 healthy employees, sedentary or moderately active (50% female and 50% male)<br>Age (mean ± SD): 49 ± 8 y<br>BMI (mean ± SD): 25.2 ± 2.5 kg/m$^2$ | Two sessions of 25-min outdoor cycling, and 20-min outdoor strength session using elastic rubber bands with handles (intensity: 55% HRR, overall)<br>**Description:** "track in a forest area nearby both workplaces. Strength | Two sessions of 25-min indoor spinning cycling, and 20-min strength session using elastic rubber bands with handles (intensity: 55% HRR, overall)<br>**Description:** "'typical' exercise setting (gym-hall), and, the subjects did not have visual contact with nature." | Affect (PAAS); BP;<br>Effort (6-20 Borg RPE scale); Enjoyment (1-item scale); Exercise behaviour (modified LTEQ); Future exercise intention (3-item scale TBP-based); HR (% of HRR); Perceived potential |

| | | | | |
|---|---|---|---|---|
| | $VO_2max$ (mean ± SD): 3.6 ± 0.8 L/min<br>HRrest (mean ± SD): 61 ± 5 bpm<br>PA (mean ± SD): 6.5 ± 4.5 h/wk<br>CNS (mean ± SD): 3.4 ± 0.6 | session took place in a grass yard" (image provided)<br>**Month:** Sept<br>**Temperature:** 8-10℃ | (image provided)<br>**Temperature:** 20℃ | for restoration (PRS); Stress (salivary CAR and serum cortisol concentration). |
| Carvalho 2010 (Sweden) | **Design:** RXT<br>Acute, 3 trials separated by at least 5-min rest<br>**Sample:** 36 post-stroke patients (31% female and 69% male)<br>Two groups based on self-selected walking speed: A: <0.8 m/s (n = 10), and B: ≥0.8 m/s (n = 26).<br>Age (mean ± SD): A, 60 ± 3 y; B, 60 ± 3 y<br>Months post-stroke (mean ± SD): A, 69 ± 43; and B, 59 ± 27<br>Assistive device (n): A, 10; B, 1. | Outdoor 6MWT at self-selected on a 30 m course and 30 m walk test at max speeds.<br>**Description:** "an outdoor walkway in a calm garden and quiet neighborhood"<br>**Temperature:** NR ("the walkway was free of snow and ice and was treated with sand") | Indoor 6MWT at self-selected and max speeds on a 30-m course in basement or clinical setting<br>**Description:** A) basement: "empty corridor" B) clinical: "corridor in a clinic rehabilitation unit".<br>"The two indoor corridors had the same regular wall-to-wall plastic carpet with an even surface, well kept, and well lit."<br>**Temperature:** NR | CRF (6MWT distance); Walking performance (self-selected and max speed over 30 m). |
| Duncan 2014 (UK) | **Design:** Non-RXT, counterbalanced<br>Acute, 2 visits separated by 24 h<br>**Sample:** 14 year 5 (ages 9-10) primary school children (50% female and 50% male)<br>Age (mean ± SD): 10 ± 1 y<br>BMI (mean ± SD): 19.2 ± 2.9 kg/$m^2$ | 15-min of cycle ergometer exercise at a moderate intensity (50% HRR and cadence of 70–80 rpm) while watching a nature video<br>**Description: "**film of cycling in a forest environment (Through the Forest; World Nature Video, Lunteren, The Netherlands)"<br>**Temperature:** NR | 15-min cycle ergometer exercise at a moderate intensity (50% HRR and cadence of 70–80 rpm) while viewing a blank screen<br>**Description:** "blank screen… Walls were blank and there were no visible windows"<br>**Temperature:** NR | BP; HR average and rest (Polar HR monitor); Mood (BRUMS). |
| Focht 2009 (USA) | **Design:** RXT, counterbalanced<br>Acute 2 single visits 48 h apart<br>**Sample:** 35 active college-age women<br>Ethnicity: 30 Caucasians, 4 African Americans, 1 Native American<br>Age (mean ± SD): 22.1 ± 1.7 y<br>BMI (mean ± SD): 22.6 ± 2.6 kg/$m^2$<br>LTEQ PA (mean ± SD): mild, 3.3 ± 2.6 | 10-min outdoor walk at a self-selected intensity (59 ± 8 % HRmax)<br>**Description:** "standardized route on sidewalks and walking paths". Clear views of nature such as grass, trees, and plants.*<br>**Temperature:** 21℃ (range: 14-28℃) | 10-min walk at a self-selected intensity (57 ± 8 % HRmax) on an indoor treadmill<br>**Description:** "laboratory setting"<br>**Temperature:** NR | Affect (FS, FAS, and EFI); Effort (Borg 6-20 RPE scale); Intention to exercise (single-item percentage scale); PA enjoyment (PACES). |

| | | | | |
|---|---|---|---|---|
| | h/wk; moderate, 3.9 ± 1.6 h/wk; strenuous, 3.5 ± 1.4 h/wk | | | |
| Fuegen 2018 (USA) | **Design:** Quasi-RCT<br>Acute,<br>**Sample:** 181 undergraduate psychology students (41 in outdoor green exercise group, 52 in indoor virtual green exercise group, 32 in outdoor resting group, and 56 in virtual green resting group) (60% female and 40% male)<br>Ethnicity: 79% Caucasian, and 9% African American<br>Age: 21.6 ± 7.69 y | 1) 15-min outdoor walk at a "comfortable pace".<br>**Description:** "route circled a lake and included views of both natural elements (i.e., trees, small plants, lake) and built elements (e.g., classroom buildings, parking lot)." (images provided)<br>**Temperature:** NR, but above 0℃, and "no evidence that variations in the weather were related to changes in mood or attention"<br>2) 15-min treadmill walk at a "comfortable, self-selected speed".<br>**Description:** "laboratory room equipped with a treadmill, computer, projector, and screen" Participants viewed via projector screen (140 cm tall · 147 cm wide) sights seen by a participant who walking along the same path as the outdoor exercise group.<br>**Temperature:** NR | No non-green exercise intervention included. | Affect (PANAS); Attention (backward digit span task and Symbol digit Modalities Test); Mood (AD-ACL) |
| Gatersleben 2013 (UK) | **Design:** RCT<br>Acute, performed 2 conditions each.<br>**Sample:** 34 students (17 in nature group and 17 in laboratory group) (59% female and 41% male)<br>Age (mean ± SD): nature group, 23.2 ± 8.2 y; laboratory group, 20.9 ± 5.0 y | 1) Two separate nature walks at participants "own speed", one in a) high prospect, low refuge (high levels of accessibility and prospect and few hiding places), and one in b) low prospect, high refuge (less accessible environments with low levels of prospect and many hiding places).<br>Average walk duration: laboratory, 9.1 | No non-green exercise intervention included. | Affect (ZIPERS); Experience of visiting country parks (1-item); Fatigue task (amended Stroop task created using E-Prime 2.0 which used both colours and shapes); Attention (NCPCT); HR (digital BP monitor). |

| | | | | |
|---|---|---|---|---|
| | | mins; nature, 10-mins.<br>**Description:** "Country park in the south of England" (images provided)<br>2) Two treadmill walk while watching 10-min videos of same walks as above (1) with background audio.<br>**Temperature:** NR | | |
| Harte 1995 (Australia) | **Design:** Non-RXT, counterbalanced<br>Acute, 4 single visits on separate days<br>**Sample:** 10 male amateur triathletes or marathon runners<br>Mean (range) age: 27 (18-37) y | 12 km run completed course on less than 45-min<br>**Description:** "12km run outdoors on a designated route around James Cook University campus"<br>**Temperature:** NR (all tests took place between 5-8am) | Two 45-min indoor runs on treadmill:<br>1) run in indoor setting with outdoor noises;<br>2) run in indoor setting with sound of heartbeat and breathing.<br>**Description:** "6 x 6m sports physiology laboratory with brick walls and high-set windows"<br>**Temperature:** NR | Attention (attention checklist); Effort (9-point Borg scale);<br>evaluation of activity;<br>Mood (POMS); Recent life events (RLEQ);<br>Systolic BP; Urinary adrenaline, noradrenaline, cortisol (expressed as a % of creatinine). |
| Irandoust 2017 (Iran) | **Design:** RCT, parallel group<br>Longitudinal, 12 weeks<br>**Sample:** 75 "severely depressed women" with Vitamin D deficiency (15 outdoor PA with vitamin D, and 15 without vitamin D; 15 indoor PA with vitamin D, and 15, without vitamin D; 15 control)<br>Age (mean ± SD): 43.2 ± 12.4 y<br>BMI (range): 30.0-35.0 kg/m$^2$ | Four 1-hour outdoor exercise sessions/week for 12 weeks. Exercise sessions: 5-min stretching, 50-min running at target HR 55-75% (Intensity progression: weeks 1-2, 55%; weeks 3-4, 65%, weeks 5-12, 75%) and RPE 11-12,, and 5-min stretching<br>**Description:** "outdoor conditions"*<br>**Temperature:** NR (sessions took place at 11-12 am in the Fall of 2015 in Qazvin, Iran) | Four 1-hour indoor exercise sessions per week for 12 weeks (same structure and intensity of outdoor condition)<br>**Description:** "treadmill walking under supervision at a health club."<br>**Temperature:** NR | 25-Hydroxyvitamin D;<br>BDI;<br>Bodyweight, BF %, BMI, and WHR. |
| Kerr 2006 (Japan) | **Design:** Non-RXT, counterbalanced<br>Acute, 2 single visits 1 week apart<br>**Sample:** 44 male students, recreational (50%) and competitive (50%) runners<br>Age (mean ± SD): recreational runners, | 5 km run at 60% HRR in natural environment<br>**Description:** "participants ran 5 km on a tree-lined footpath… outdoor running pathway ran alongside two | 5 km indoor run at 60% HRR on a treadmill<br>**Description:** "treadmill located in the university sports medicine laboratory" | Emotional response (TESI). |

| | | | | |
|---|---|---|---|---|
| | 22.7 ± 1.7 y, and competitive middle and long distance runners, 20.6 ± 1.3 y | small lakes, through woods and playing fields and the road had only occasional traffic."<br>**Temperature:** NR ("weather and ground conditions were similar (sunny, no wind, dry ground)") | **Temperature:** NR ("Temperature and humidity conditions in the laboratory were similar to conditions outside") | |
| Lacharite-Lemieux 2015 (also 2016) (Canada) | **Design:** RCT, parallel groups<br>Longitudinal, 12 weeks<br>**Sample:** 23 healthy, sedentary postmenopausal women divided into two groups (11, indoor, and 12, outdoor)<br>Age (mean ± SD): indoor, 59.4 ± 3.8 y; and outdoor, 62.0 ± 5.5 y<br>BMI (mean ± SD): indoor, 25.7 ± 1.8 kg/m$^2$; and outdoor, 25.4 ± 1.9 kg/m$^2$<br>PASE PA (mean ± SD): indoor, 148.0 ± 75.2 kcal/wk; and outdoor, 148.3 ± 54.5 kcal/wk<br>Environmental preference (indoor/outdoor): indoor, 5/6; and outdoor, 5/7 | Three weekly 1-hour mixed aerobic (10-min: 'performing movements' and 20-min: circuit training; intensity 65%-95% HRmax) and resistance training (15-min) sessions conducted in outdoor natural park **Description:** "mainly natural park beside a body of water, where paths were lined with large trees and rich biodiversity... river and some old pine trees."<br>**Months**: Apr-Jul **Temperature:** NR ("On days with heavy rain, the group exercised under the large tent located in the center of the park near the water.") | Three weekly 1-hour mixed aerobic (10-min: 'performing movements' and 20-min: circuit training; intensity 65%-95% HRmax) and resistance training (15-min) conducted in indoor room<br>**Description:** "Research Center on Aging… The floor was carpeted and the room had many windows, with a view of the parking lot on both sides."<br>**Temperature:** NR ("participants in both groups experienced the same temperature conditions on hot days… indoor participants avoided the cold days of early April.") | Affect (EFI; FS; FAS); BMI, fat mass, LBM, and muscle mass index (DXA); BP ( automatic BP monitor); CRF (via modified Balke test); Depressive symptoms ( BDI); Effort (via Borg 6-20 RPE); Exercise adherence (% of sessions attended); Fasting glucose, insulin, and plasma lipids; HR (Polar HR monitor); Muscular endurance (max number of repetitions at 70% 1RM); Muscular strength (1RM test); PA (PASE). |
| McMurray 1988 (USA) | **Design:** Non-RXT<br>Acute, 2 single visits on separate days<br>**Sample:** 8 male runners<br>Age (range): 21-41 years<br>Weight (mean ± SEM): 70.2 ± 1.3 kg<br>$VO_2$max (mean ± SEM): 59 ± 3 ml/kg/min | 10 mile outdoor run (same intensity as indoor run)<br>**Description:** "outdoors on a predetermined, fairly level, 10 mile course" (First and final laps were performed on a 400m athletics track)<br>**Temperature** (mean ± SD)**:** 25 ± 2℃ (slight breeze)<br>**Humidity** (mean ± SD)**:** 65 ± 6 % | 10 mile run on indoor treadmill at speed and grade setting equal to 70% VO2 max<br>**Description:** Indoor "laboratory" setting<br>**Temperature** (mean ± SD)**:** 22 ± 1℃ (cooling fans were used for all runs)<br>**Humidity** (mean ± SD)**:** 40 ± 12 % | Affect (GAS); VO2 (via open circuit spirometry and Douglas Bags);<br>HR (via ECG and palpitation);<br>Plasma beta-endorphin and lactate concentrations (via blood samples). |

| | | | | |
|---|---|---|---|---|
| Mieras 2014 (USA) | **Design:** RXT, counterbalanced<br>Acute, 2 sessions (no fewer than 2 days and no longer than 2 weeks apart)<br>**Sample:** 12 recreationally trained male cyclists<br>Ethnicity: 11 Caucasian, 1 African-American<br>Age (mean ± SD): 37 ± 2 y<br>Weight (mean ± SD): 82.1 ± 4.8 kg<br>BF % (mean ± SD): 15.1 ± 1.8 %<br>$VO_2$max (mean ± SD): 53 ± 2 ml/kg/min | 40 km outdoor cycling on set course at self-selected intensity (consistent effort)<br>**Description:** "outdoor trials were completed along a relatively flat, out and back course on a paved recreation trail (Keystone Trail, Omaha, NE, USA)."<br>**Month**: Aug-Oct<br>**Temperature** (Mean ± SD): 22.1 ± 0.2℃<br>**Humidity** (Mean ± SD): 32.0 ± 1.4%<br>**Wind speed** (Mean ± SD): 2.5 ± 0.6 m/s<br>Heat Index, Dew point, wet bulb, and pressure similar between conditions | Laboratory cycling on 40 km at self-selected training intensity (consistent effort)<br>**Description:** "Exercise physiology laboratory, where environmental conditions remain relatively constant"<br>**Temperature** (Mean ± SD): 22.0 ± 0.1 ℃<br>**Humidity** (Mean ± SD): 38.3 ± 3.4%<br>**Wind speed** (Mean ± SD): 0.0 ± 0.0 $m/s^{-1}$ | Attentional focus (via TAF scale); Bodyweight; Core temperature (CBTC); Effort (Borg 6-20 RPE scale); HR; Performance power output (power meter); Skin temperature (Thermostor patch); USG (via digital refractometer). |
| Niedermeier 2017a (also 2017b) (Austria) | **Design:** RXT<br>Acute 3 single sessions with mean time between sessions of 1 week (1-14 day range)<br>**Sample:** 42 healthy adults (48% female and 52% male)<br>Age (mean ± SD): 32 ± 12 y<br>BMI (mean ± SD): 23.0 ± 2.0 $kg/m^2$<br>PA (mean ± SD): 8 ± 5 h/week<br>Mountain tours (mean ± SD): 27.2 ± 26.2 tours/year | 3-h of outdoor mountain hiking in groups of five (6 km of uphill walking in ~1.5 h to 1500m at average speed 4 km/h; 10-min rest; walking downhill for 70-min at average speed 5.2 km/h<br>**Description**: "famous hiking area and started at the northern edge of Innsbruck with direct access to natural environment."<br>**Month:** May, Aug, Sept/Oct<br>**Temperature:** NR | 3-h of indoor treadmill walking in groups of five (6 km of walking uphill on inclination of 10% for 1.5 h at 4 km/h; 10-min rest; 70-min of level walking at average speed 5.2 km/h<br>**Description:** "situated in a fitness centre."<br>**Temperature:** NR | Affect (FS, FAS); Affective states (MSS & STAI); BP; Effort (Borg 6-20 RPE scale); HR (Polar HR monitor); HRV; Salivary cortisol. |

| | | | | |
|---|---|---|---|---|
| Nisbet 2011 (Canada) | **Design:** RCT x 2<br>Acute, single session in both study 1 and 2.<br>**Sample:** Study 1) 150 University students (57% female, 37% male, and 6% unspecified)<br>Age (mean ± SD): 20.8 ± 5.0 y<br>Study 2) 80 participants (no participant characteristics were reported) | 1) 17-min walk in nature in groups of 1-11 students.<br>**Description:** "walking and biking path along the Rideau Canal… a green corridor… 8 km through the heart of Ottawa... relatively picturesque… urban nature."<br>2) Participants received description of walk and rated their anticipated effect before walking in a different outdoor environment to above<br>**Description:** "on-campus… walking path between a road and a river that borders the campus."<br>**Temperature:** 2.5-14.6°C ("Walks conducted in Fall on days with no rain.") | 1) 17-min walk in indoor environment in groups of 1-11 students<br>**Description:** "proceeded to their destination, the athletics building, via tunnels"<br>2) Participants received description of walk & rated their anticipated effect before they walked in an indoor environment<br>**Description:** "different building than the indoor route in Study 1, but also used parts of the university tunnel system"<br>**Temperature:** NR | Affect (PANAS); Nature Relatedness (INS scale); Relaxation, Fascination, curiosity, and interest (modified PANAS scale). |
| Peacock 2007 (UK) | **Design:** Non-RXT<br>Acute 2 single visits 1 week apart<br>**Sample:** 20 participants (65% female and 35% male)<br>Age range: 31-70 y (aged 31-50 y: 47%; aged 51-70 y: 53%) | 30-min outdoor walk. No intensity provided but "continuous walking was preferred... participants were allowed to stop briefly to admire the scenery… social interaction was also encouraged"<br>**Description:** "Belhus Woods Country Park, which has a diverse landscape of woodlands, grasslands and lakes."<br>**Temperature:** NR | 30-min indoor walk. No intensity provided but "continuous walking was preferred... participants were allowed to stop briefly to admire… shop windows, and a certain level of social interaction was also encouraged"<br>**Description:** "walking around Lakeside shopping centre."<br>**Temperature:** NR | Enjoyment (via 1-5 Likert scale); Mood (POMS); Self-esteem (RSE scale). |
| Plante 2003 (USA) | **Design:** RCT, parallel groups<br>Acute, 4 single visits on separate days<br>**Sample:** 154 undergraduate psychology students (66% female and 34% male)<br>Age: NR<br>BMI (mean ± SD): outside, women 21.1 ± 2.7 kg/m$^2$ and men 24.6 ± 3.7 kg/m$^2$; | 1) 20-min brisk (~3 mph) outdoor walk.<br>**Description:** "Garden at the campus of Santa Clara University"*<br>2) 20-min VR with walking (speed: 2.7-3.5 mph) in laboratory. | 20-min of walking on a treadmill (speed: 2.7-3.5 mph) without any VR technology<br>**Description:** "No visual stimulus was presented before the walk to the participants"<br>**Temperature:** NR | Mood (AD-ACL); Social Desirability or defensiveness (MC-SDS) |

| | | | | |
|---|---|---|---|---|
| | laboratory, women 23.5 ± 3.3 kg/m$^2$ and men 24.1 ± 2.3 kg/m$^2$ | **Description:** "same route around campus that walkers used in condition 1 (see above)" (sunny conditions)<br>**Temperature:** NR ("no participant was made to walk in rainy weather or at night.") | | |
| Plante 2006 (USA) | **Design:** RCT, parallel groups<br>Acute, 2 single visits on separate days<br>**Sample:** 112 undergraduate psychology students (58% female and 42% male) (no participant characteristics were reported) | 1) 20-min brisk outdoor walk (speed ~4.8 km/h)<br>**Description:** "Garden at the campus of Santa Clara University"*<br>2) 20-min walking (speed: 4.3–5.6 km/h) on a laboratory treadmill while watching a video projected on a screen of the same route around campus that walkers in condition 1 (see above) completed (sunny day)<br>**Temperature:** NR ("northern California during the fall… no participant was made to walk in rainy weather or at night.") | No non-green exercise intervention included. | Mood (AD-ACL); PA enjoyment (PACES); Social Desirability or defensiveness (MC-SDS). |
| Plante 2007 (USA) | **Design:** RCT, parallel groups<br>Acute, single visit<br>**Sample:** 88 female undergraduate students<br>Age (mean ± SD): 19 ± 1 y | 1) 20-min moderate intensity walk (60-70% HRmax) along a prescribed route on the university campus.<br>2) Same walk as above but accompanied by a friend<br>All conditions performed "same exercise task in terms of type and intensity of exercise."<br>**Description:** "Garden at the campus of Santa Clara University"*<br>**Temperature:** NR | 1) 20-min moderate intensity walk (60-70% HRmax) alone on a treadmill.<br>2) Completed the same treadmill walk as above but did so alongside a friend who walked on an adjacent treadmill.<br>**Description: "**University fitness facility on campus"<br>**Temperature:** NR | Mood (AD-ACL); PA enjoyment (PACES). |
| Rider 2016 (Canada) | **Design:** RXT, counterbalanced<br>Acute, 2 10-minute walks | 10-minute (~0.5 km) nature walk (intensity not reported) | 10-minute (~0.5 km) indoor walk (intensity not reported) **Description:** | Memory (Free recall test, Forced-choice recognition |

| | | | | |
|---|---|---|---|---|
| | Study 1) participants studied a word list, then walked; Study 2) participants walked, then studied a word list.<br>**Sample:** Study 1) 24 undergraduate students (83% female and 17% male)<br>Mean age: 22 y<br>Study 2) 24 undergraduate students (79% female and 21% male)<br>Mean age: 22 y | **Description:** "walk through a number of stands of trees, bushes, and grassy areas along a relatively quiet asphalt path on University campus." (image provided)<br>**Month:** Sept-Oct 2014<br>**Temperature:** -5°C to 21°C (weekday mornings or afternoons) | **"**hallways on three floors of the University building and did not provide much exposure to natural or urban outdoor elements"<br>**Temperature:** -5°C to 21°C | test); Mood (11-point scale, -5 = very negative, 0 = neutral, +5 = very positive); Rating of experience (asked to indicate which walk was most enjoyable, most beautiful, least distracting). |
| Rogerson 2016 (UK) | **Design:** RXT, counterbalanced Acute, 3 visits - baseline, indoors, outdoors (baseline to first condition separated by 6 ± 3 d; condition 1 and 2 separated by 9 ± 8 d). Participants took part in pairs (who already knew each other)<br>**Sample:** 24 participants (79% female and 21% male) (1 staff member, 10 students, 13 public) Age (mean ± SD): 35.1 ± 20.1 y Weight (mean ± SD): 70 ± 15 kg | 15-min on cycle ergometer at 50% HRR. Participants advised that they were free to talk as much or as little as they liked.<br>**Description: "**Exercise was performed outside, on the University sports fields, a large area of largely level gradient, maintained grassland, lined and partly interspersed with trees" (Image provided)<br>**Temperature:** NR | 15-min on cycle ergometer at 50% HRR. Participants advised that they were free to talk as much or as little as they liked.<br>**Description:** Exercise was performed in a laboratory (8.3 x 4.9 m). with a view of a white painted brick wall (Image provided)<br>**Temperature:** NR | Directed attention (backward digit span task); Effort (Borg RPE); Mood (30-item POMS); Intention for future (VAS [0-100] to rate enjoyment of session, intention to attend a session in the future—if offered free); Social interaction time. |
| Rogerson 2015 (UK) | **Design:** RXT, counterbalanced<br>Acute, 4 visits separated by 7-25 days (average: 13 days)<br>**Sample:** 12 healthy adult participants (50% female and 50% male)<br>Age (mean ± SD): 27.8 ± 5.5 y<br>Weight (mean ± SD): 65.4 ± 10.5 kg | 1) 15-min bout of exercise on the treadmill, at 60% VO2 peak (via gas analysis) while watching nature video 2) run at 85% VO2 peak to voluntary exhaustion while watching nature video<br>**Description:** video consisted of scenes extracted from "Evening Run through Endless Forest". (all videos played at the same speed without auditory sound)<br>**Temperature:** NR (time of day was consistent between occasions-within 2 hours) | 1) 15-min bout of exercise on the treadmill, at 60% VO2 peak (via gas analysis) while watching 1) blank screen and 2) watching built environment video<br>2) run at 85% VO2 peak to voluntary exhaustion while watching 1) "blank white screen" and 2) watching built environment video<br>**Temperature:** NR | Directed Attention (subtraction of serial sevens test, spelling words backwards, and backwards digit span test); Effort (Borg 6-20 RPE scale); Energy expenditure ((gas analysis method not reported)); HR average; Respiratory exchange ratio (gas analysis method not reported); TTE at 85% VO2 peak. |

| | | | | |
|---|---|---|---|---|
| Ryan 2010 (USA) | **Design:** RCT, parallel groups (study 2)<br>Acute, single visit<br>**Sample:** 80 undergraduate university students (82% female and 18% male)<br>Mean (range) age: 20 (18-22) y | 15-min walk in outdoor setting (no intensity reported)<br>**Description:** "Participants in the outdoor condition walked on a largely tree-lined footpath along a river that runs parallel to the university campus."<br>**Month:** Sept-Oct<br>**Temperature:** NR (walks completed 11am-4pm) | 15-min walk in indoor setting (no intensity reported)<br>**Description:** "series of underground hallways and tunnels that were devoid of living things"<br>**Temperature:** NR (walks completed 11am-4pm) | State vitality (SVS) |
| Teas 2007 (USA) | **Design:** Non-RXT<br>Acute 2 single visits 1 week apart<br>**Sample:** 19 healthy, non-smoking, postmenopausal women<br>Ethnicity (n): European American, 13; African American, 6<br>Age (mean ± SD): 58 ± 4 y<br>BMI (mean ± SD): 27 ± 6 kg/m$^2$ | 1-hour walk at a "comfortable" self-chosen speed (mean: 5.1 km/h) outdoors<br>**Description:** "campus horseshoe (grassy area lined with brick paths, old trees, and flowerbeds)."<br>**Month:** May<br>**Temperature:** 22°C (women walked together on same day at 6:20-7:20 pm)<br>**Humidity:** 45%<br>(Light: 220 lux; Noise: 65 decibels) | 1-hour walk at a "comfortable" self-chosen speed (mean: 4.3 km/h) on a treadmill<br>**Description:** "gym lab was located in the university gym, and provided an environment similar to that found in a commercial gym"<br>**Temperature:** 23°C (walk completed 5:00-6:45pm)<br>**Humidity:** 46%<br>(Light: 180 lux; Noise: 74 decibels) | Negative affect (NAS); Positive affect (PAS); Salivary cortisol and alpha amylase |
| Turner 2017 (UK) | **Design:** RXT, counterbalanced<br>Acute, 2 visits at least 24 hours apart<br>**Sample:** 22 adult competitive and recreational runners (36% female and 64% male)<br>Age (n): 18-34 y, 14; 35-51 y, 8<br>BMI (n): ≤ 24.9 kg/m$^2$, 16; ≥ 25.0 kg/m$^2$, 6<br>Relatedness to nature (n): high, 19; low: 3 | 6 km run - first 3 km at steady-state pace, second 3 km at maximum intensity (fast as possible) with the second half completed at maximum effort, followed by 10-min recovery<br>**Description: "**Large woodland area, with walking/running trails lined with trees and bushes... Dog walkers and other runners were present."<br>**Temperature:** 17.5°C<br>(typically cloudy with sunny intervals) | 6 km run - first 3 km at steady-state pace, second 3 km at maximum intensity (fast as possible) with the second half completed at maximum effort, followed by 10-min recovery<br>**Description:** Treadmill in a large fitness suite. "Digital screens on the treadmill displaying feedback data remained visible, to allow for self-pacing. Other users of the fitness suite were present".<br>**Temperature:** 19°C | Affect (FS, FAS); Effort (Borg 6-20 RPE scale); State vitality (7-item SVS). |

| | | | | |
|---|---|---|---|---|
| White 2015 (UK) | **Design:** RXT<br>Acute, 4 visits ~1 week apart<br>**Sample:** 37 postmenopausal women<br>Age (mean ± SD): 50.1 ± 3.7 y<br>BMI (mean ± SD): 25.3 ± 4.7 kg/m$^2$<br>Self-reported instances of at least 30-min of light-moderate exercise per week (mean ± SD): 4.5 ± 2.9 instances. | Cycle ergometer for 15-min while watching a 1) green video and 2) blue video.<br>**Description:** Green video: 3 x 5-min scenes of fields with sheep, hedgerows and a small wood. Blue video: 3 x 5-min clips from a headland overlooking a beach and of views from beach height across rocks and the sea<br>**Temperature:** NR | Cycle ergometer for 15-min while facing 1) a blank wall (control) or 2) watching an 'urban video'.<br>**Temperature:** NR | Affect (FS, FAS); BP; Effort (Borg RPE scale); Experience (Enjoyment of session); HR; Time perception (asked participants for how long they felt they had been cycling). |
| Yeh 2017 (UK) | **Design:** Non-RXT, counterbalanced<br>Acute, 3 20-min treadmill runs with minimum 7 day gap between conditions<br>**Sample:** 30 adults (40% female and 60% male)<br>Age (mean ± SD): 28 ± 9 y<br>BMI (mean ± SD): 22.2 ± 2.1 kg/m$^2$ | 1) 20 minute treadmill run at self-selected pace looking at static image of nature (visual only)<br>2) 20 minute treadmill run at self-selected pace looking at dynamic image of nature (visual only)<br>**Description:** Dynamic image condition: video of the "Sheffield Botanical Gardens… series of paths within the gardens, capturing the trail through lawns, trees and flower beds… sunny spring afternoon"<br>**Temperature:** NR | 20 min treadmill at self-selected pace focusing on self-selected entertainment (music = 23; movies with sound = 6; viewing a picture = 1)<br>**Description:** "self-selected, preferred entertainment where participants were able to choose preferences that included visual and/or auditory information"<br>**Temperature:** NR | Affect: happiness, anxiety, dejection, anger, and excitement (SEQ); Distance (via treadmill);<br>HR (Polar HR watch). |

**Key:** RXT: Randomised crossover trial; BMI: Body Mass Index; RCT: Randomised comparative trial; VR: virtual reality; $VO_2max$/peak: maximal/peak oxygen uptake; CVD: cardiovascular disease; HR: heart rate; HRV: Heart Rate Variability; PA: physical activity; CRF: Cardiorespiratory Fitness; SF: short form; BP: blood pressure; LTEQ: Godin's Leisure Time Exercise Questionnaire; CNS: Connectedness to Nature Scale (1-5 scale, higher the score, greater the connectedness to nature); PAAS: Physical Activity Affective Scale; PRS: Perceived Restorativeness Scale; CAR: Cortisol Awakening Response; 6MWT: 6-min walk test; BRUMS: Brunel Mood State Inventory; FS: Feeling Scale; FAS: Felt Arousal Scale; EFI: Exercise-Induced Feeling Inventory; GAS: General affect scale; PACES: Physical Activity Enjoyment Scale; ZIPERS: Zuckerman Inventory of Personal Reactions; NCPCT: Necker Cube Pattern Control task; RPE: Rate of Perceived Exertion; DXA: dual energy X-ray absorptiometry; SVS: Subjective Vitality Scale; PAS: Positive Affect Scale; NAS: Negative Affect Scale; WHR: Waist-Hip Ratio; BF: body fat; TESI: Tension and Effort Stress Inventory; RSE: Rosenberg Self-esteem; POMS: Profile Of Mood States; RLEQ: Recent Life Events Questionnaire; AD-ACL: Activation–Deactivation Adjective Check List; TTE: time to exhaustion; PANAS: Positive and Negative Affect; Schedule; MC-SDS: Marlowe-Crowne Social Desirability Scale; TAF: Tammen Attentional Focus; PASE: Physical Activity Scale for the Elderly; CBTC: Core Body Temperature Capsule; USG: Urine Specific Gravity; INS: Inclusion of Nature in Self; SEQ: Sport Emotion Questionnaire; 1-RM (one repetition-maximum).

**Table S2.** Outcomes from longitudinal studies not included in the meta-analysis

| Outcomes | Calogiuri 2015 | Irandoust & Taheri 2015 | Lacharite-Lemieux 2015 |
|---|---|---|---|
| Anthropometric outcomes | | Statistical pre to post-intervention reductions in:<br>• Waist-to-hip ratio in exercise groups only (*p*'s>.05). | No statistical time by environment interaction effect for:<br>• Waist circumference<br>• Fat mass (via dual x-ray absorptiometry [DXA])<br>• Lean body mass (via DXA) |
| Psychological outcomes | Statistically higher:<br>• Perceived restorativeness of the environment (fascination and being away, *p*'s<.001);<br>• Enjoyment (after outdoor biking, *p*=.02; overall enjoyment, *p*>.05);<br>• Intention to exercise in the future after correcting for previous exercise behaviour (*p*<.01). | | No statistical changes as a function of time or environment in:<br>• Revitalization;<br>• Fatigue scores. |
| Exercise adherence, intensity, and physical fitness | Statistically higher:<br>• Adherence to exercise in the outdoor versus indoor exercise groups (97% vs. 91% of prescribed sessions completed).<br><br>No statistical differences in:<br>• Average and maximal heart rates during exercise (bpm and %, *p*'s≥.05);<br>• Cardiorespiratory fitness (via estimated $VO_2max$. (However, a statistical pre to post-intervention increase with indoor exercise, *p*=.01), but not outdoor exercise, *p*=.082) | | No statistical time by environment interaction for upper (bench press and lat pulldown) and lower (leg press) body muscular strength.<br>• Both green and indoor exercise statistically improved lower body strength (*p*=.016 and *p*=.021, respectively);<br>• Only green exercise statistically improved upper body strength (bench press, *p*=.006; lat pulldown, *p*=.017)<br>• Both green and indoor exercise improved leg press endurance from |

| | | | |
|---|---|---|---|
| | | | pre to post-intervention ($p<.001$ and $p=.001$, respectively)<br>• Muscular endurance at bench press ($p=.003$) and lat pulldown ($p=.023$) only increased with green exercise. |
| Biological biomarkers | Statistical improvements in green exercise vs. indoor exercise:<br>• Salivary cortisol awakening response (CAR) area under the curve with respect to increase (AUCI) in the ($p=.04$).<br><br>No between group differences in:<br>• CAR area under the curve with respect to the ground;<br>• Serum cortisol concentration. | Statistically higher serum 25-hydroxyvitamin D concentrations with:<br>• Green exercise plus vitamin D supplementation vs. indoor exercise with vitamin D ($p<.05$);<br>• Green exercise vs. indoor exercise without vitamin D ($p<.05$). | No statistical changes with either green or indoor exercise in:<br>• Fasting glucose and insulin,<br>• Homeostasis Model Assessment-insulin resistance (HOMA-IR);<br>• Triglycerides;<br>• Total, low-density lipoprotein, and high-density lipoprotein cholesterol. |

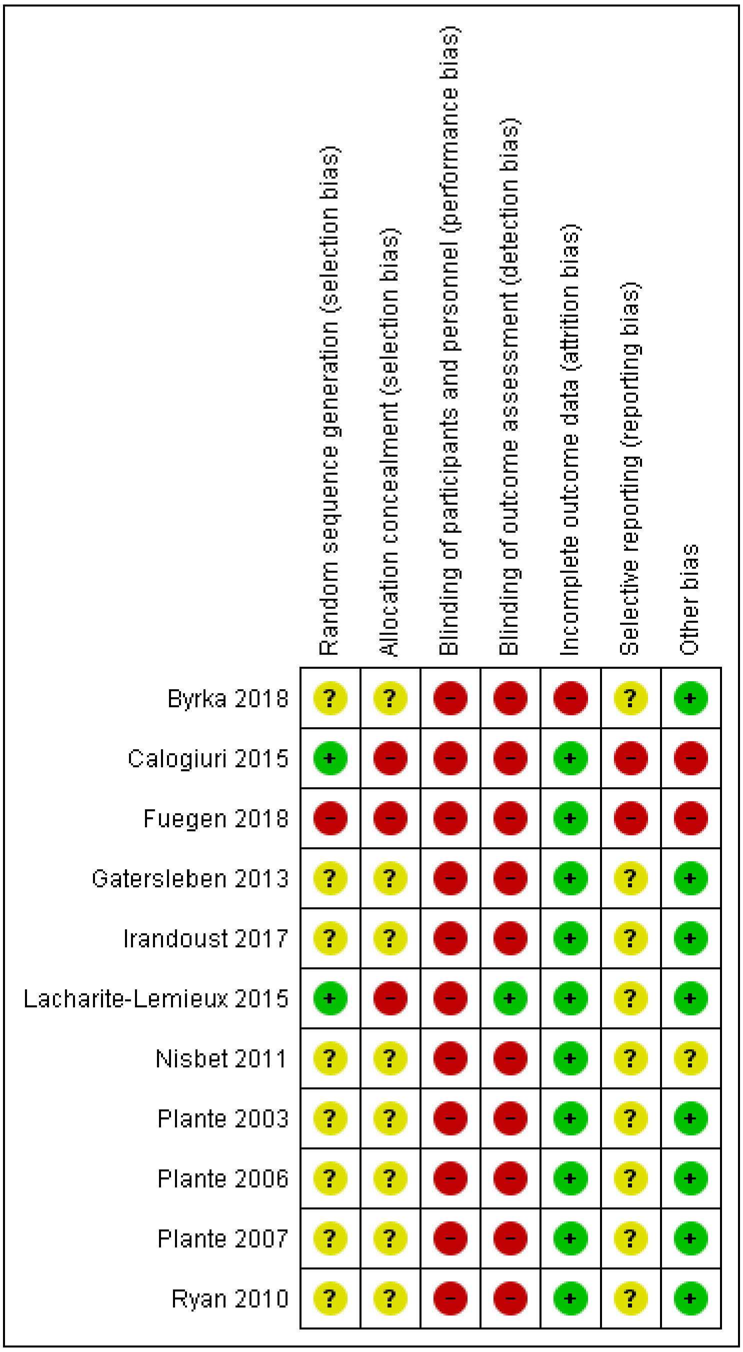


**Figure S1.** Risk of bias summary across comparative trials: review authors' judgements about each risk of bias item for each included study.

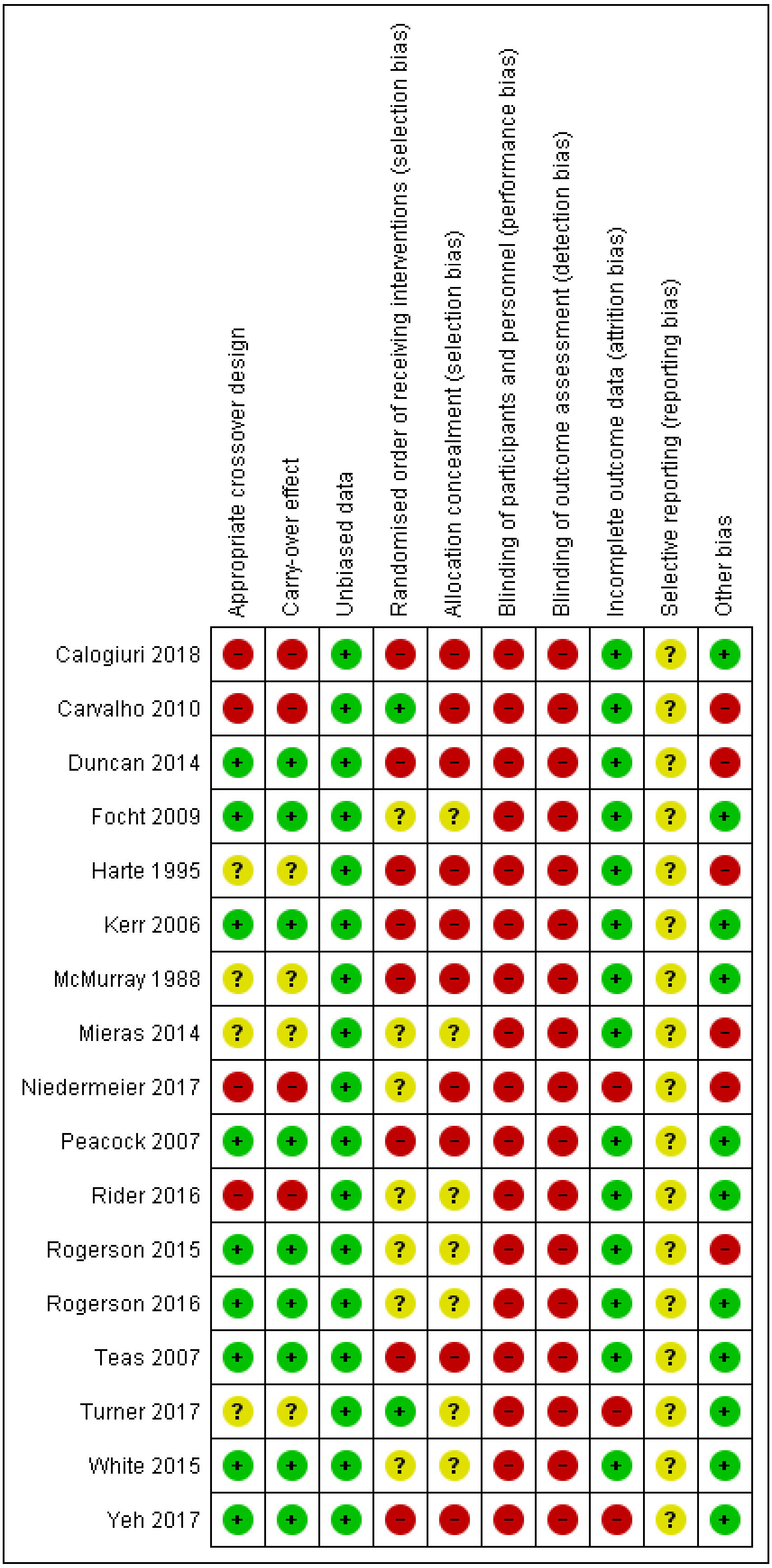


**Figure S2.** Risk of bias summary across crossover trials: review authors' judgements about each risk of bias item for each included study.
